# Assessment of the consistency and robustness of results from a multicenter trial of remission maintenance therapy for acute myeloid leukemia

**DOI:** 10.1186/1745-6215-12-86

**Published:** 2011-03-23

**Authors:** Marc Buyse, Pierre Squifflet, Kathryn J Lucchesi, Mats L Brune, Sylvie Castaigne, Jacob M Rowe

**Affiliations:** 1 International Drug Development Institute, Department of Biostatistics, Louvain-la-Neuve, Belgium; 2 I-BioStat, Center for Statistics, Hasselt University, Diepenbeek, Belgium; 3 MedVal Scientific Information Services, Medical Writing Department, Skillman, NJ, USA; 4 Sahlgrenska Academy, University of Gothenburg, Hematology Unit, Gothenburg, Sweden; 5 Hôpital André Mignot, Service Hématologie et Oncologie, Le Chesnay, France; 6 Rambam Medical Center, Department of Hematology and Bone Marrow Transplantation, and Technion, Haifa, Israel

## Abstract

**Background:**

Data from a randomized multinational phase 3 trial of 320 adults with acute myeloid leukemia (AML) demonstrated that maintenance therapy with 3-week cycles of histamine dihydrochloride plus low-dose interleukin-2 (HDC/IL-2) for up to 18 months significantly improved leukemia-free survival (LFS) but lacked power to detect an overall survival (OS) difference.

**Purpose:**

To assess the consistency of treatment benefit across patient subsets and the robustness of data with respect to trial centers and endpoints.

**Methods:**

Forest plots were constructed with hazard ratios (HRs) of HDC/IL-2 treatment effects versus no treatment (control) for prospectively defined patient subsets. Inconsistency coefficients (I^2^) and interaction tests (X^2^) were used to detect any differences in benefit among subsets. Robustness of results to the elimination of individual study centers was performed using "leave-one-center-out" analyses. Associations between treatment effects on the endpoints were evaluated using weighted linear regression between HRs for LFS and OS estimated within countries.

**Results:**

The benefit of HDC/IL-2 over controls was statistically consistent across all subsets defined by baseline prognostic variables. I^2 ^and *P*-values of X^2 ^ranged from 0.00 to 0.51 and 0.14 to 0.91, respectively. Treatment effects were statistically significant in 14 of 28 subsets analyzed. The "leave-one-center-out" analysis confirmed that no single center dominated (*P*-values ranged from 0.004 to 0.020 [mean 0.009]). The HRs representing the HDC/IL-2 effects on LFS and OS were strongly correlated at the country level (R^2 ^= 0.84).

**Limitations:**

Small sample sizes in some of the subsets analyzed.

**Conclusions:**

These analyses confirm the consistency and robustness of the HDC/IL-2 effect as compared with no treatment. LFS may be an acceptable surrogate for OS in future AML trials. Analyses of consistency and robustness may aid interpretation of data from multicenter trials, especially in populations with rare diseases, when the size of randomized clinical trials is limited.

**Trial Registration:**

ClinicalTrials.gov: NCT00003991

## Introduction

The results of a clinical trial should not be assessed solely in terms of statistical significance. In their *Statistical Principles for Clinical Trials *(ICH E9), the International Conference on Harmonization recommends evaluating "*the robustness of the results and primary conclusions of the trial. Robustness is a concept that refers to the sensitivity of the overall conclusions to various limitations of the data, assumptions, and analytic approaches to data analysis*" [1]. Hence a trial that reached the standard criterion of significance (*P *< 0.05) could still be questioned if its results lacked robustness. In contrast, when studying diseases of low incidence, achieving *P *< 0.05 may require sample sizes that are too large to be achievable in a reasonable timeframe. Estey argues that if a disease is relatively uncommon and active therapies are lacking, protection against false positive results with >95% confidence may be too stringent [2]. This point is clearly illustrated by acute myeloid leukemia (AML), a disease with incidence ranging from 2 to 4 per 100,000 persons in Europe and the United States [3]. Trials that aim to demonstrate statistically significant benefits on overall survival (OS) in AML are especially challenging since they require large numbers of patients and long durations of follow-up. With typical costs and time to conduct oncology trials in excess of $500 million and 10 years, respectively [4], new approaches to study design and interpretation that help reduce these burdens are obviously necessary. Intensive efforts are therefore underway to evaluate other approaches to bring promising new drugs that fulfill urgent medical needs to patients more efficiently [4-9].

First and foremost, when evaluating new cancer treatments, a number of efficacy endpoints are usually considered. In both early and advanced disease, commonly used endpoints are OS and disease or progression-free survival (DFS or PFS) [6-8]. In the case of acute leukemia, if a trial reaches statistical significance on DFS (more commonly called leukemia-free survival, LFS) but not on OS, is it because the treatment actually has an effect on one endpoint but not on the other, or merely because the effect seen on LFS is attenuated in the analysis of OS? In fact, attenuation of the treatment effect on OS is expected because of three independent factors: (a) the time lag between leukemia recurrence and death, which results in a lower hazard ratio for OS than for LFS for the same absolute number of events; (b) variations in post-relapse therapies that may have effects on OS completely unrelated to the treatment being evaluated [10], and (c) competing risks of death that may be substantial in a disease such a AML, for which the median age at diagnosis is approaching 70 years [11,12]. Hence in AML, both LFS and OS are important, the former because it is statistically sensitive to real treatment effects, and the latter because it is the ultimate endpoint that cancer treatment should affect. Therefore, an investigation of the relationship between LFS and OS can be informative, in addition to analyses of each endpoint considered separately.

Second, if an overall treatment effect of a novel therapy is detected, it is of interest to understand whether the benefit applies to all patients, or if the benefit is confined to particular patient subsets. This is especially relevant in a heterogeneous disease such as AML, which is comprised of small groups of patients with distinctly different prognoses determined by age, karyotype, cytogenetics, and level of minimal residual disease, among others [2,5]. Such prognostic information is already being used to direct therapeutic decision-making, and this trend will undoubtedly increase as more cytogenetic information about AML becomes available [2]. In this respect, a study of the consistency of the treatment effects across subsets of patients based on prognostic variables can provide useful information to clinicians.

Third, if an overall treatment effect is detected in a multicenter trial, what assurance can be made that the effect is not heavily influenced by a single center or very few centers? In a multinational trial, are the efficacy outcomes and the treatment effects on these outcomes broadly comparable between countries? A study of the robustness of the treatment effects with respect to centers, and of the consistency of these effects with respect to countries, can provide assurance that the trial results are broadly representative and, as such, more likely to be generalizable.

This paper addresses these issues in the context of a randomized multinational phase 3 trial of histamine dihydrochloride, used in conjunction with low-dose interleukin-2 (HDC/IL-2) as remission maintenance therapy in AML patients [13]. This trial achieved statistical significance on LFS (the pre-specified primary endpoint) and showed that treatment with HDC/IL-2 prolonged LFS compared to controls (standard-of-care; no treatment). Although the sample size was substantial for a trial in AML (320 patients, 236 AML relapses, 196 deaths), the trial was insufficiently powered to detect an effect on OS and did not reach statistical significance on the OS endpoint. In this paper, we show that analyses of consistency and robustness can help interpret these results.

## Methods

### Phase 3 clinical trial of HDC/IL-2 as maintenance therapy for AML patients in complete remission

This was a randomized open-label trial of 320 AML patients in complete remission (CR), post-induction and consolidation treatment. The trial was conducted according to ethical principles stated in the Declaration of Helsinki (October, 1996). The protocol, amendments, and sample informed consent forms were reviewed and approved at each of 92 distinct clinical centers by a duly constituted Institutional Review Board or Independent Ethics Committee [13]. Each patient was required to read, understand, sign and date a copy of the informed consent form in the presence of the investigator (or designee) before any protocol-specified procedures were undertaken.

A large proportion of patients were in first complete remission (CR1; n = 261). Patients who received an allogeneic transplant during first remission were ineligible. Immunotherapy with HDC (Ceplene^®^, EpiCept Corporation, Tarrytown, NY) was given subcutaneously (sc) at a dose of 0.5 mg BID in conjunction with IL-2 (Proleukin^®^, Chiron, Emeryville, CA [now Novartis]) 16,400 IU/kg sc BID. Following initial supervision and training to perform sc injections, treatments were self-administered by patients for up to 10 × 3-week cycles over a maximum period of 18 months. Control patients received no treatment during this period.

The primary objective of this trial was to determine if HDC/IL-2 could prolong LFS compared with no treatment. LFS was defined as the number of days from the date of randomization to the date of relapse of AML or death from any cause, whichever came first. Relapse was determined by examination of the bone marrow using an identical schedule of clinical and laboratory assessments in both treatment arms. The effect of HDC/IL-2 on OS was a secondary endpoint. Hazard ratios (HRs) for LFS and OS were estimated with a Cox regression model with treatment as the covariate of interest (coded 1 = treatment, 0 = control, so that HR>1 indicates treatment benefit), and stratification for country and complete remission (CR1 vs CR>1).

In this trial, HDC/IL-2-treated and untreated groups were well balanced across all demographic and disease prognostic variables [13]. A significant benefit of HDC/IL-2 was demonstrated for the primary LFS endpoint (HR = 1.43, *P *= 0.008), but not for OS (HR = 1.23, *P *= 0.16). Treatment with HDC/IL-2 was well-tolerated, with no treatment-related mortality, significant morbidity, or detrimental impact on quality-of-life [14]. Details about the trial and its major results have been previously reported [13,14]. Treatment with HDC/IL-2 was approved in Europe in October 2008 for AML patients in CR1. The analyses presented herein are by intention-to-treat (ITT) on all randomized patients. A level of 0.05 was used throughout as the nominal threshold of statistical significance, keeping in mind that *P*-values of individual tests must be interpreted with due allowance for multiplicity.

### Consistency of treatment effects

Forest plots of LFS HRs were constructed for all prognostic subsets thought to be relevant at the time the study was conducted. Forest plots were also constructed for the countries in which patients were treated; the United Kingdom, Finland, and Estonia had included only 7 patients in total and were not shown on the plots.

Tests of heterogeneity (X^2^) and inconsistency coefficients (I^2^) were used to assess the observed differences in LFS HRs among the various subsets [15,16]. The test statistic for heterogeneity between S subsets, X^2^, is defined as X^2 ^= Σ (τ_i _- τ)^2^/s_i_^2^, where τ_i _is the treatment effect in the i^th ^subset, s_i _is the standard error of τ_i_, and τ is the overall treatment effect. X^2 ^has a χ^2 ^distribution with (S - 1) degrees of freedom [15]. The inconsistency index between S subsets, I^2^, is calculated as I^2 ^= (X^2 ^- S + 1)/X^2 ^if X^2 ^> S - 1, and I^2 ^= 0 otherwise [16]. I^2 ^values indicate little inconsistency if they are under 0.33, moderate inconsistency if they range from 0.33 to 0.67, and substantial inconsistency if they are above 0.67 [16].

When a subset showed a negative treatment effect (i.e. patients in the control group fared better than patients in the treatment group), we calculated the probability that such a reversal of effect could be observed just by the play of chance [17]. To calculate an approximate probability, we observe that if the N patients of the trial are subdivided in S subsets of equal size, the standard error of the subset-specific test statistic is equal to **√**S times the standard error of the overall test statistic. The probability of a reversal of effect is given by the area under the normal distribution of the subset-specific test statistic to the left of zero. Note that for a time to event endpoint such as LFS or OS, the "size" of a subset is its number of events. Reference [17] provides further details in the general case.

### Robustness of treatment effects

The trial was conducted in 92 distinct clinical centers with the number of patients treated within these centers ranging from 1 to 17. The majority of centers had too few patients to yield informative estimates of treatment effects. Therefore, a "leave-one-center-out" cross-validation was performed to assess the robustness of HDC/IL-2 effects with respect to site. *P*-values for treatment effect were re-calculated after sequential elimination of individual study centers and summarized as a frequency distribution. In addition, *P*-values for treatment effect were re-calculated after successive elimination of the largest and the smallest centers from the analysis in order to estimate the number of such eliminations required in order to lose statistical significance.

### Association between treatment effects on different endpoints

In order to investigate the consistency of outcomes and of treatment effects, we used the approach developed for the validation of surrogate endpoints [18,19]. This approach consists of quantifying the associations between the two endpoints (LFS, the potential surrogate, and OS) and between the treatment effects on the two endpoints [9]. These analyses are described in detail in a separate manuscript. Here, we focused on the association between treatment effects and fitted a weighted linear regression between the HRs for LFS and OS estimated within countries. Coefficients of determination (R^2^) were calculated to quantify the proportion of variance explained by the regressions.

## Results

### Consistency of treatment effects across patient characteristics and countries

**Figure 1:**
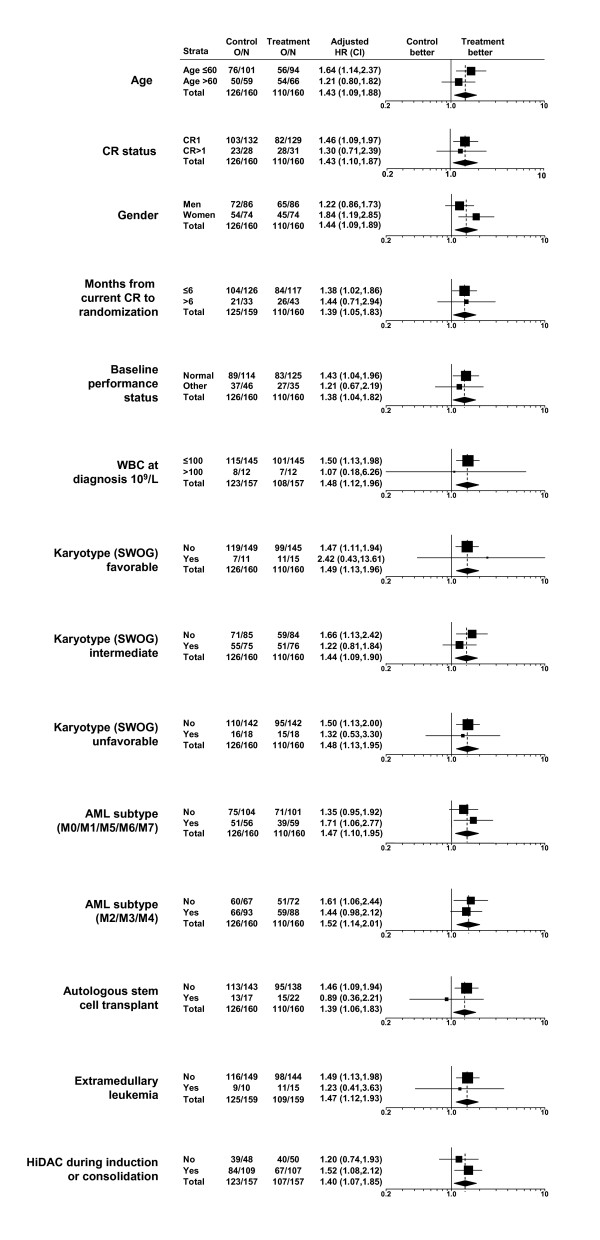
**Forest plots of leukemia-free survival (LFS) hazard ratios (HR) and their confidence intervals (CI) by baseline characteristics**. O/N = event rate per arm where O is the number of observed events (relapse or death) and N is the sample size. HR = hazard ratio, CI = confidence interval, CR = complete remission, CR1 = first complete remission, WBC = white blood cell, SWOG = Southwest Oncology Group, AML = acute myeloid leukemia, HiDAC = high-dose cytosine arabinoside.

Compared with no treatment, HDC/IL-2 had a statistically significant benefit on LFS (HR = 1.43, 95% CI = 1.10, 1.87, log-rank test stratified for country and CR status *P *= 0.008) [13]. Figure 1 shows forest plots of LFS HRs in all randomized patients and in subsets based on baseline patient characteristics: age (> or ≤60 years), CR status (first [CR1] or subsequent remission [CR >1]), gender, months from CR to randomization (> or ≤6 months), performance status, white blood cell counts at diagnosis, Southwest Oncology Group (SWOG) karyotype, AML subtype, intensity of prior induction and consolidation therapy (autologous stem cell transplant or high dose cytarabine), and presence of extramedullary leukemia. Adjusted for country and CR status, HRs reflecting treatment benefit exceeded 1.00 in 27 subsets out of 28 examined, with statistical significance detected in 14 of 28 subsets analyzed, indicating consistency across subsets.

Testing for interactions to determine whether the benefit of HDC/IL-2 differed among these subsets, X^2 ^values were all non-significant and ranged from X^2^_1d.f. _= 0.01 to X^2^_1d.f. _= 2.06. The X^2 ^for country was also non-significant, whether the 7 countries with 313 patients were considered (X^2^_6d.f. _= 9.63, *P *= 0.14) or whether all 10 countries were included (X^2^_9d.f. _= 9.86, *P *= 0.36). Most I^2 ^values were either equal to 0 or lower than 0.33, except for gender (I^2 ^= 0.51) and country (7 countries, I^2 ^= 0.38). The moderate inconsistency noted for gender and country could not be explained either through confounding factors, or through some other prior information.

**Figure 2:**
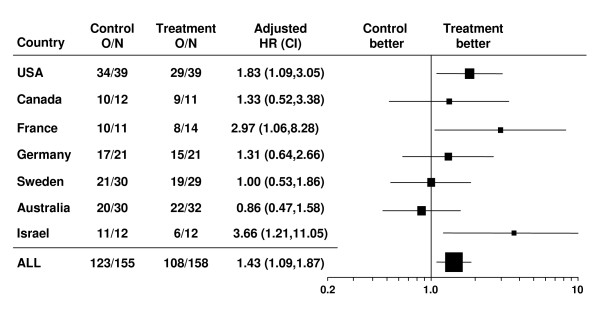
**Forest plots of leukemia-free survival (LFS) hazard ratios (HR) and their confidence intervals (CI) by country**. O/N = event rate per arm where O is the number of observed events (relapse or death) and N is the sample size.

Country-specific HRs stratified by CR-status were larger than 1.00 in 5 out of 7 countries included in these analyses (Figure 2), with statistical significance reached in three of them, indicating that the results were not driven by a single influential country. The treatment effect was negative in one country, but with 7 countries of equal size such a reversal of effect would be expected to occur just by chance with probability 0.16, which is close to one in seven.

### Robustness of treatment effects

**Figure 3:**
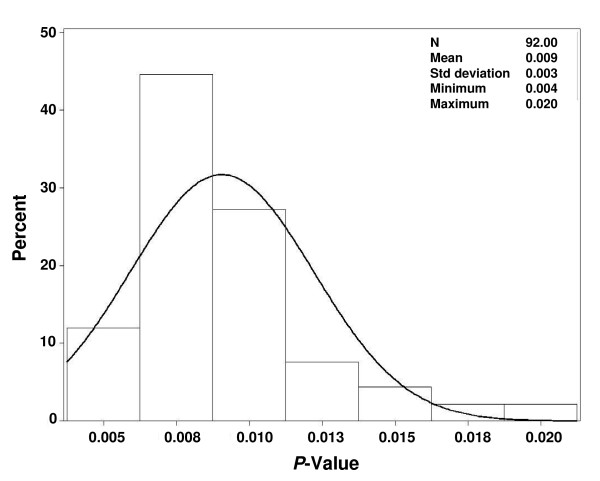
**Distribution of *P*-values for the treatment effect on leukemia-free survival in a "leave-one-center-out" cross-validation**. N = sample size.

The "leave-one-center-out" cross-validation performed with the 92 distinct centers yielded *P*-values ranging from 0.004 to 0.020 (mean 0.009) for the LFS analysis, instead of *P *= 0.008 (for the observed LFS hazard ratio of 1.43). Hence, the *P*-value for treatment effect remained significant after elimination of any study center (Figure 3), thereby providing confidence that no study center was so influential as to drive the statistical significance of the findings.

When *P*-values for treatment effect were re-calculated after successive elimination of several centers from the analysis, statistical significance was retained until elimination of the 8 largest centers (HR = 1.32, *P *= 0.084, 83 patients eliminated), and the 29 smallest centers (HR = 1.32, *P *= 0.052, 35 patients eliminated).

### Association between treatment effects on different endpoints

**Figure 4:**
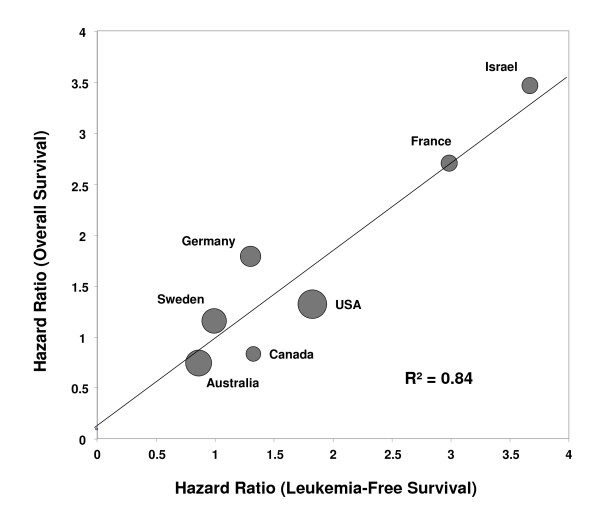
**Correlation between treatment effects on leukemia-free survival and overall survival (R^2 ^= coefficient of determination)**. The size of each circle is proportional to the number of patients in the corresponding country.

Country-specific HRs reflecting the treatment effects on LFS and OS were highly correlated (Figure 4). The weighted linear regression equation was HR_OS _= 0.10 + 0.86 × HR_LFS _with a coefficient of determination R^2 ^= 0.84, indicating that 84% of the variance was explained by the linear regression (*P *= 0.004). Hence, the observed effect of treatment on LFS was a good predictor of the effect of treatment on OS, with only a slight (14%) attenuation of the effect as reflected in the slope of 0.86. Additionally, the fitted regression line passed nearly through the origin, indicating that no effect on LFS would predict no or little effect on OS (as expected).

## Discussion

We critically inspected the results of this randomized phase 3 trial of HDC/IL-2 as remission maintenance therapy versus no treatment for AML patients in complete remission. Treatment effects were assessed for consistency and robustness with respect to clinically relevant prognostic variables and with respect to center or country where the treatment took place. To this end, we tested for treatment by subset interactions (Table 1 and Figures 1 and 2), calculated inconsistency indices (Table 1), performed leave-one-center-out cross-validation (Figure 3), showed that several centers could be eliminated before losing statistical significance and correlated country-specific treatment effects on LFS and on OS (Figure 4). Taken together, these analyses indicate that the benefit of HDC/IL-2 is statistically consistent and robust.

**Table 1:** Heterogeneity test statistics (X^2^) and inconsistency coefficients (I^2^) for baseline disease characteristics, corresponding to the hazard ratio forest plots of Figures 1 and 2

Baseline disease characteristic	**X**^**2 **^**(*P*-value)**	**I**^**2**^
Age (≤60 vs >60)	1.19 (0.28)	0.16
CR status (CR1 vs CR >1)	0.12 (0.73)	0.00
Gender (Men vs Women)	2.06 (0.15)	0.51
Months from current CR to randomization (≤6 vs >6)	0.01 (0.91)	0.00
Performance status (Normal vs Other)	0.23 (0.63)	0.00
WBC at diagnosis (10^9^/L) (≤100 vs >100)	0.13 (0.71)	0.00
Karyotype (SWOG): Favorable (No vs Yes)	0.31 (0.58)	0.00
Karyotype (SWOG): Intermediate (No vs Yes)	1.16 (0.28)	0.14
Karyotype (SWOG): Unfavorable (No vs Yes)	0.07 (0.79)	0.00
AML subtype: M0/M1/M5/M6/M7 (No vs Yes)	0.60 (0.44)	0.00
AML subtype: M2/M3/M4 (No vs Yes)	0.14 (0.71)	0.00
Autologous stem cell transplant (No vs Yes)	1.03 (0.31)	0.03
Extramedullary leukemia (No vs Yes)	0.12 (0.73)	0.00
High dose of cytarabine received (No vs Yes)	0.63 (0.43)	0.00
Country (7 countries)	9.63 (0.14)	0.38
Country (10 countries)	9.86 (0.36)	0.09

CR = complete remission, CR1 = first complete remission, SWOG = Southwest Oncology Group.

By "consistent," we mean that (a) the treatment effects do not differ by more than random variation across prognostic factors and other design features such as country; and (b) the treatment effects on different endpoints are highly correlated. By "robust," we mean that (a) the treatment effects would have been about the same had slightly different patient populations been included (this aspect of robustness derives directly from the consistency of the results); and (b) the treatment effects remain significant even after elimination of a few centers from the analysis. We suggest that similar assessments could be useful in all randomized multicenter trials aimed at establishing the efficacy and safety of new therapies, particularly in trials with limited sample sizes (eg, resulting from a low incidence of the disease under study). These analyses would complement other sensitivity analyses that are recommended to examine the influence of protocol deviations, unintended biases, violations of assumptions and other unexpected events on the trial outcome [1].

It is commonly believed that homogeneity of the patient population through narrow selection criteria is a desirable feature of phase 3 clinical trials. This is because heterogeneity will tend to increase the variance in patient outcomes, thereby reducing the likelihood of real treatment effects reaching statistical significance. In fact, the opposite is true insofar as heterogeneity across patient and disease characteristics at baseline renders the results of the trial more generalizable. Moreover, if patients with widely different baseline characteristics are included, potential treatment-by-prognostic-factor interactions can be found. To this end, inconsistency indices, which are commonly used in meta-analyses [16], may prove more descriptively useful than interaction tests that generally lack power to detect any but the most extreme interactions.

Another commonly held view is that having a large number of sites, as is the case in most cancer trials, somehow reduces the credibility of the findings. Here again, the opposite is true. When a trial is able to show a statistically significant difference despite the presumed heterogeneity resulting from the multicentric nature of patient accrual, the trial results are even more convincing, as well as more generalizable, than if all patients came from a few carefully selected centers. An assessment of the robustness of the trial results may be useful regardless of the number of centers participating in a trial, but they are more likely to be convincing if a large number of centers participated in the trial, as was the case in the trial analyzed in the present paper.

Although there is little question that the results of this trial were consistent and robust, the point estimate of the treatment effect was zero (no effect) in one country, and negative (control better than treatment) in another. Marschner [17] argues that such findings are often overinterpreted, and proposes ways to assess the expected variability in country-specific treatment effects at the design stage. We showed that a post-hoc calculation of the probability of a reversal of the treatment effect (under some simplifying assumptions) can be useful to address concerns that there is variability in treatment effect between countries, over and above chance alone. Such analyses may be especially relevant when potential predictive factors are suspected to vary by region, perhaps as a result of genetic differences related to ethnicity.

When evaluating therapies for cancer, many factors can influence the ability to detect a statistically significant survival benefit. In the case of AML, a relatively uncommon disease, enrollment of patients in large enough numbers to adequately power a study for OS is a major challenge. Second, long follow-up durations are required, during which practice patterns change and impact OS in ways extraneous to the treatment effect. Third, most AML patients are older and have a higher probability of death than younger patients (5-year survival rates are 4% and 31% in persons ≥65 and <65 years of age, respectively) [20] and such deaths in older patients unrelated to leukemia have the potential to confound interpretation of OS data [10]. Fourth, with particular relevance to the study of remission maintenance therapies in AML, patients may receive salvage therapies post-relapse. Post-relapse salvage therapies are far from standardized and have widely different mortality risks; hence, any observed differences in OS might result from such therapies rather than from the randomized intervention. For these reasons, LFS may be more appropriate than OS to assess the benefit of strategies to prevent AML relapse.

In the present trial, with the available follow-up data at the time of the analysis, 236 patients had experienced an event contributing to the LFS endpoint (110 in the treatment group and 126 in the control group) and 196 patients had died (94 in the treatment group and 102 in the control group). Hence, the power of the LFS analysis was higher than that of the OS analysis and it was expected, for this reason only, that a higher level of significance would be reached for LFS than for OS. With this in mind, it seemed useful to assess the correlation between the effects of treatment on LFS and OS to better understand whether treatment with HDC/IL-2 was likely to have a real effect on OS, regardless of its (lack of) statistical significance. We have explored this issue using countries as the unit of analysis, extending methods that were initially proposed for meta-analyses of several trials [18].

## Conclusions

Our analyses confirm the consistency and robustness of the HDC/IL-2 effect as compared with no treatment. LFS may be an acceptable surrogate for OS in future AML trials. Similar analyses may aid interpretation of data from multicenter trials, especially in populations with rare diseases, when the size of randomized clinical trials is limited.

## Abbreviations

AML: acute myeloid leukemia, BID: twice daily, CI: confidence interval, CR: complete remission, CR1: first complete remission, CR>1: subsequent complete remission, d.f.: degrees of freedom, DFS: disease-free survival, Σ: summation, HDC: histamine dihydrochloride (Ceplene^®^), HiDAC: high dose cytosine arabinoside, HR(s): hazard ratio(s), I^2^: inconsistency coefficient, ICH: International Conference on Harmonization, IL-2: interleukin-2, ITT: intent-to-treat, **√**S: square root of S, LFS: leukemia-free survival, M0/M1/M5/M6/M7 and M2/M3/M4 refer to the French-American-British AML classification, O/N: event rate per arm where O is the number of observed events and N is the sample size, OS: overall survival, P: probability, PFS: progression-free survival, R^2^: coefficient of determination, S: number of subsets, s_i_: standard error of τ_i_, SWOG: Southwest Oncology Group, τ: overall treatment effect, τ_i_: treatment effect on the i^th ^subset, WBC: white blood cell, X^2^: interaction test for homogeneity, χ^2^: chi-square

## Competing interests

MB is majority shareholder of IDDI and PS is a Biostatistician at IDDI, a company that provides biostatistical services to EpiCept Corporation. KJL is a Senior Medical Writer at MedVal Scientific Information Services and a consultant for EpiCept Corporation. MLB, SC, and JMR were investigators on the EpiCept-sponsored study of HDC/IL-2 and have received honoraria from EpiCept Corporation.

## Authors' contributions

MB, PS, MLB, SC, and JMR made substantial contribution to conception and study design. MB, PS, MLB, SC, and JMR were involved in analysis and interpretation of data. MB, PS, KJL, MLB, SC and JMR were involved in drafting or revising the manuscript for important intellectual content. MB, PS, KJL, MLB, SC and JMR read and approved the final manuscript for publication.
